# Non-Vitamin K Oral Anticoagulants Assessment in High Risk of Bleeding Patients with Non-Valvular Atrial Fibrillation

**DOI:** 10.3390/geriatrics7010020

**Published:** 2022-02-17

**Authors:** Pedro Silva Cunha, André Viveiros Monteiro, Madalena Coutinho Cruz, Paula Malveiro, João Pedro Reis, Guilherme Portugal, Ana Dias, Rui Cruz Ferreira, Mário Martins Oliveira

**Affiliations:** 1 Arrhythmology, Pacing and Electrophysiology Unit, Cardiology Service, Santa Marta Hospital, Central Lisbon Hospital University Center, 1169-024 Lisbon, Portugal; andreviveirosmonteiro@chlc.min-saude.pt (A.V.M.); madalena.cruz@chlc.min-saude.pt (M.C.C.); paula.malveiro@chlc.min-saude.pt (P.M.); joao.pedro.reis@chlc.min-saude.pt (J.P.R.); guilherme.portugal@chlc.min-saude.pt (G.P.); ana.dias@chlc.min-saude.pt (A.D.); rui.ferreira@chlc.min-saude.pt (R.C.F.); mario.martins.oliveira@chlc.min-saude.pt (M.M.O.); 2 Faculty of Medicine, University of Lisbon, 1649-028 Lisbon, Portugal

**Keywords:** non-vitamin K oral anticoagulants, atrial fibrillation, anticoagulation unit

## Abstract

Atrial fibrillation (AF) is commonly associated with advanced age and the presence of multiple, concomitant acute and chronic health conditions, placing this population at high risk for serious therapeutic side effects. Nonvitamin K antagonist oral anticoagulants (NOACs) are increasingly used for stroke prevention in patients with atrial fibrillation. The purpose of this study was to investigate the effectiveness and safety of NOAC in a group at high risk of bleeding complications, in a real-world setting. We conducted a retrospective analysis of a high-risk cohort of 418 patients (pts) followed-up in our anticoagulation unit; data on patient characteristics, anticoagulation treatment, and bleeding and thrombotic complications were evaluated. The population had a median age of 77.8 ± 10.3 years and the mean CHA_2_DS_2_-VASc score was 3.85 (SD ± 1.4). Overall, 289 (69.1%) were ≥75 years old. During a mean follow-up time of 51.2 ± 35.7 months, we observed a rate of any bleeding of 7, a clinically relevant non-major bleeding rate of 4.8, a major bleeding rate of 2.2, a stroke rate of 1.6, and a rate of thrombotic events of 0.28 per 100 patient-years. There were 59 hospitalizations due to any cause (14.1%) and 36 (8.6%) deaths (one due to ischemic stroke). A structured follow-up, with judicious prescribing and drug compliance, may contribute to preventing potential complications.

## 1. Introduction

Atrial fibrillation (AF) is a highly prevalent arrhythmia; its incidence increases with advancing age, and it is considered a significant cause of mortality worldwide [1,2]. The most serious consequence of AF is the occurrence of thromboembolic events, particularly stroke [3]. The risk of thromboembolic events increases with the presence of factors such as advanced age, ventricular dysfunction, arterial hypertension, female gender (when associated with other risk factors), and cardiovascular disease (previous myocardial infarction, arterial disease, or aortic plaques), as well as the previous occurrence of stroke [4,5]. In this context, one of the most important aspects of the treatment of patients with AF is the prevention of thromboembolic events. 

Although the clinical benefit of anticoagulants outweighs potential risks, there can be significant disadvantages to anticoagulant therapy, including cost, and, most importantly, an increased risk of potentially life-threatening bleeding complications [6]. The annual incidence of major bleeding among individuals with AF on oral anticoagulation varies widely, ranging from 1.3% to 7.2%. [7]. Of particular concern is the risk of intracranial hemorrhage (ICH), which is associated with high rates of mortality and morbidity. 

Patients with AF form a heterogeneous group with a variety of clinical characteristics and risk factors for bleeding and/or thromboembolism. Several factors affect bleeding risk, including the intensity of anticoagulation, the efficacy of monitoring modalities, and patient characteristics [8].

As the prevalence of atrial fibrillation increases with age, it augments the risk of embolic stroke in elderly individuals; however, oral anticoagulant therapy in elderly patient is largely underutilized [9], despite the net clinical benefit demonstrated in those patients [10]. In the ATRIA study, warfarin was used in approximately 60% of patients aged 65–84 years and only 35% of those aged ≥85 years, among elderly individuals with nonvalvular atrial fibrillation and no contraindications [11]. Moreover, the incidence of frailty among patients with AF is on the rise, which indicates that patients with AF are more prone to frailty compared to patients without AF [10]. The presence of frailty predicts poorer outcomes and decreased anticoagulation use in patients with nonvalvular atrial fibrillation [9]. As previously mentioned, AF is commonly associated with advanced age (≈70% of AF patients are 65 to 85 years old and 10% are ≥80 years) [12] and also with the presence of multiple concomitant acute and chronic health conditions. For this reason, it is estimated that AF patients have as high as 4-fold increased odds of being classified as frail compared with other patients [13]. Frailty definitions in broad patient populations have included several clinical parameters (age, nutritional deficits, decreased mobility, social withdrawal, low income, number of prior hospitalizations, and cognitive impairment) [14]. Specifically, in the context of bleeding risk, anticoagulation trials have defined frailty as the presence of age >75 years old, creatinine clearance < 50 mL/min, or bodyweight ≤ 50 kg [15].

Non-vitamin K antagonist oral anticoagulants (NOAC) are easier to use and might offer similar or better levels of stroke prevention with a similar or reduced risk of bleeding, which should increase the use of antithrombotic therapy in the management of elderly AF patients [16]. The safety profiles showed that all NOACs caused a lower risk of intracranial hemorrhage, but an increased risk of gastrointestinal bleeding with rivaroxaban, edoxaban, and dabigatran (150 mg twice daily) compared with warfarin [17,18].

European guidelines [19] have expressed a preference for NOACs over vitamin K antagonists (VKAs) in stroke prevention for non-valvular AF patients, especially if newly initiated. NOACs have been well accepted in clinical practice, as they are considered to have predictable pharmacokinetics, a lack of food interactions, and fewer drug interactions, allowing for standardized dosing without monitoring. However, adherence to therapy remains a concern [20], and their misuse could potentially result in patient harm, particularly in the group with the high-risk profile, like the elderly and patients with renal impairment [21,22].

In our country, there is a paucity of data evaluating the non-vitamin-K oral anticoagulants (NOACs) in patients treated in routine practice. Therefore, we sought to investigate NOAC dosing patterns and the effectiveness and safety of a structured follow-up of high-risk-of-bleeding patients in the framework of a real-world setting NOAC anticoagulation unit.

## 2. Methods

We conducted an observational, single-center, retrospective cohort study in patients with AF treated with a NOAC for stroke prevention, who were followed up in the anticoagulation outpatient clinic, and who accomplished more than 12 months of follow-up. Declaration approval of the Institutional Board Review was obtained (CES 974/2020). All participants provided written informed consent for data collection and the study was conducted in accordance with the Declaration of Helsinki.

Data from patients followed-up between May 2016 and June 2020 were collected for each patient from clinical registries and included demographics, clinical information, height, weight, renal function, pharmacological therapy, date of initiation of therapy, presence of comorbidities, cardiovascular, hemorrhagic, or thromboembolic events, as well as follow-up data until the last appointment at the unit, or death. 

Patients were all referred by the outpatient clinic of the Cardiology Department due to high-risk clinical characteristics, namely the presence of advanced age, high CHADS-VASC score, and/or chronic renal dysfunction, and all were treated with the NOACs currently available (dabigatran, rivaroxaban, apixaban, edoxaban). The indication at the commencement of therapy was stroke prevention in the context of atrial fibrillation in all patients. The standard operating procedures for this unit follow the European Heart Rhythm Association Practical Guide and involve checking visit adherence, thromboembolic events, bleeding events, other side effects, co-medications, blood sampling to calculate glomerular filtration rate, plasmatic urea and creatinine levels, modifiable risk factors, and optimal NOAC selection and dosing.

At the first appointment in the anticoagulation unit, the adequacy of dosage was evaluated by the physician, and the patient received information regarding anticoagulation in general and received an in-depth description about the use of a NOAC from the nurse, which included bleeding risks and information regarding routine laboratory assessments. All patients received an “anticoagulation card”, with hospital contacts, which identifies the diagnosis that indicates anticoagulation, as well as the commercial name of the prescribed anticoagulant and the respective dosage.

The primary outcome was the occurrence of any bleeding reported during long-term follow-up, based on the definition of the International Society on Thrombosis and Hemostasis [23]. Accordingly, major bleeding is defined as fatal bleeding, and/or bleeding in a critical area or organ (intracranial, intraspinal, intraocular, retroperitoneal, intra-articular or pericardial, or intramuscular with compartment syndrome), and/or bleeding causing a fall in hemoglobin level of 20 g/L or more, or leading to transfusion of two or more units of whole blood or red cells. Clinically relevant nonmajor bleeding was defined [24] as overt bleeding that does not fit the criteria for the ISTH definition of major bleeding but required medical intervention by a healthcare professional, leading to hospitalization or increased level of care, or prompting a face-to-face (i.e., not just a telephone or electronic communication) evaluation. 

The secondary outcomes were the occurrence of stroke/transient ischemic attack (TIA), venous thromboembolic complications (pulmonary embolism, deep venous thrombosis), systemic arterial embolism (e.g., renal infarction or low-extremity artery occlusion), hospital admission, and death.

### Statistical Analysis

Descriptive analysis was performed to characterize the cohort data. Mean and standard deviation (mean ± SD) measures were used to summarize continuous variables. Absolute and relative frequencies expressed as percentages (%) are presented for categorical data. Logistic regression analysis was employed to assess the impact of various factors as predictors of hemorrhagic and thrombotic events, and death. IBM SPSS Statistics^®^ version 24 (IBM SPSS Statistics for Windows, Armonk, NY: IBM Corp.)was used for the statistical analysis. All *p* values were 2-sided, and values <0.05 were considered to indicate statistical significance.

## 3. Results

### 3.1. Study Population 

**Figure 1:**
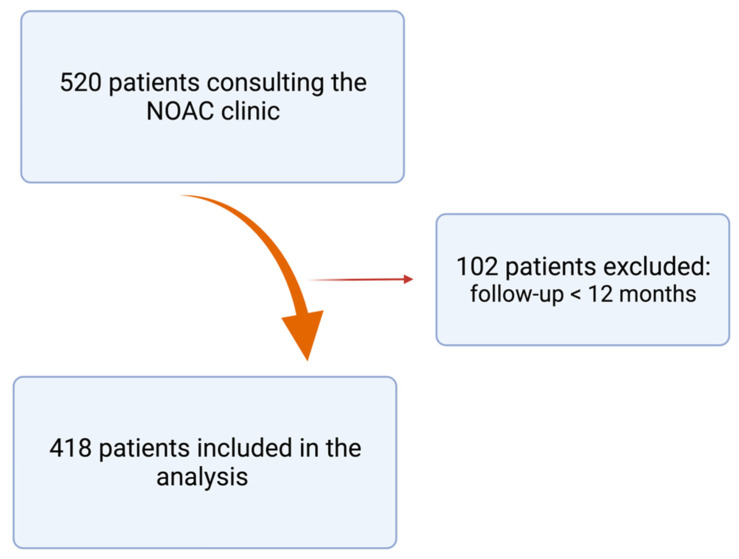
Flowchart of patient inclusion. NOAC non-vitamin- K oral anticoagulant; 102 patients were excluded due to a follow-up of fewer than 12 months.

Out of a population of 520 patients followed-up at our outpatient anticoagulation unit, we identified 418 patients (Figure 1) with high-risk bleeding characteristics (advanced age and renal failure) and follow-up ≥ 12 months [25]. In this cohort, the median age was 77.8 ± 10.3 years, and 54.5% (*n* = 228) were male patients. A large proportion of patients (*n* = 289; 69.1%) were ≥75 years old (of these, 220 patients were aged ≥80 years, and 30 were above 90 years old). The mean follow-up time was 51.2 ± 35.7 months.

Mean glomerular filtration rate at NOAC initiation was calculated according to the CKD-EPI 2009 formula, with impaired renal function observed in 89.7% of the population (GFR < 89, stage

≥2

of renal function). From this chronic kidney dysfunction (CKD) sample, 46.6% had moderate (stage 3) CKD, and 9.8% had severe CKD.

The mean CHA_2_DS_2_-VASc score was 4.32 ± 1.18 for those aged ≥75 years and the mean HAS-BLED score was 2.1 ± 1 for those aged ≥75 years. Detailed results are presented in Table 1.

Regarding the distribution of medication (Table 2), the most frequently prescribed NOAC was apixaban, used in 182 patients (43.4%), followed by edoxaban (*n* = 136; 32.6%), rivaroxaban (*n* = 82; 19.5%), and dabigatran (*n* = 16; 3.7%) (Table 2). A significant percentage of the population (*n* = 187; 44.7%) was under a reduced dose of the NOAC, reasons for this adjustment being: the presence of one or a combination of ≥2 factors (depending on the NOAC), including age ≥80 years, the presence of renal dysfunction, weight

≤

60 kg and concomitant use of ketoconazole. The percentage of patients with concomitant medication with antiplatelet agents was only 1.4%.

### 3.2. Follow-Up

During follow-up, a total of 3665 outpatients visits were analyzed. Bleeding was documented in 50 patients (11.9%)—including clinically relevant non-major bleeding in 34 patients (8.1%) and major bleeding in 16 patients (3.8%)—of which the large majority (80%) of bleeds were in patients ≥75 years old. Stroke/transient ischemic attack was reported in 12 patients (2.6%), 91% aged ≥75, and thrombotic events were observed in two patients during follow-up.

These data translate to a rate of any total bleeding events (major and non-major clinically relevant bleeding) (Table 3) of 7 events per 100 patient-years. In addition, the clinically relevant non-major bleeding rate was 4.8 per 100 patient-years, the major bleeding rate of 2.2 per 100 patient-years, the stroke rate was 1.6 per 100 patient-years, and the rate of thrombotic events was 0.28 per 100 patient-years. The number of hospitalizations (due to any cause) was 59 (14.1%), all in patients ≥75 years old. 

Mortality data were available in 100% of patients; during follow-up 36 (8.6%) deaths occurred (one due to ischemic stroke), a rate of 5 per 100 patient-years.

A temporary interruption of NOACs was disclosed in 87 patients (20.8%) (Table 4), mainly due to medical interventions, namely surgery (*n* = 50), endoscopy (*n* = 20), dental extraction (*n* = 7), or biopsy (*n* = 5).

In the univariate logistic regression analysis, age, weight, CHADS-VASC, and HAS-BLED scores, creatinine, and glomerular filtration rate (both at baseline and during follow-up) were not associated with the combined endpoint of thrombotic or hemorrhagic events, hospitalization, and death.

## 4. Discussion

Our retrospective study provides real-world, long-term data on the use of NOACs in non-valvular AF in high-risk-for-bleeding patients followed in an anticoagulation unit. 

Although NOACs do not require the meticulous dose adjustments required for warfarin, a clinical evaluation of appropriate doses remains necessary [26,27].

There is an inverse correlation between prescription of oral anticoagulation (OAT) and elderly patients [28], being age an independent predictor of non-prescription. The reasons for the underuse of anticoagulants in the elderly are essentially related to the fear of bleeding. In patients aged ≥75 years, the incidence of AVK bleeding increases up to 5% per year. Compared to patients aged 70–79 years, the risk of bleeding does not increase in patients of age 80–89 years and only increases by 26% in patients aged >90 years [6,29]. Intracranial hemorrhages are 2.5 times more frequent in patients over 85 years, and account for about 90% of deaths or lead to severe disability among survivors. The higher bleeding risk with the VKAs led to the underuse of oral anticoagulant therapy in the elderly, but the main advantage of using NOACs over AVKs was the reduction of intracranial hemorrhages [9].

The large proportion of elderly patients in our sample (69.1% ≥75 years) and the high-risk nature of their conditions, with a mean CHA_2_DS_2_-VASc score of 3.85, and 89.7% with impaired renal function (46.6% moderate (stage 3) CKD; 9.8% severe CKD) reflect a high level of clinical complexity, with CKD rates higher than the findings from previous reports [30,31,32].

Recently Chao [33] has shown that in patients with AF and age ≥90 years OAT is associated with a low risk of ischemic stroke and an obvious net clinical benefit, and NOACs are associated with a low risk of intracranial hemorrhage. The authors emphasize the choice of NOACs for thromboprophylaxis in the very old. 

Coleman’s study group [34], showed that the use of rivaroxaban, but not apixaban or dabigatran, is associated with a reduction in stroke and systemic embolism versus warfarin in frail elderly patients. In addition, rivaroxaban treatment reduces lesions in frail elderly patients with venous thromboembolism, thromboembolic recurrence, and has a better impact on bleeding compared to warfarin. Evidence shows different reproducibility rates of the data from DOACs clinical trials (both phase IV studies and “real-life” ones) above all, in terms of safety. The incidence of frailty can largely account for the differences in performance. A meta-analysis from Ruff et al. [35] involving 71,683 patients with AF from the registration trials pointed out the significant reduction in stroke and systemic embolism incidence (relative risk (RR), 0.81; confidence interval (CI) 95%, 0.73–0.91; *p* < 0.0001) in NOACs patients as compared to warfarin, as well as all-cause mortality (RR, 0.90; CI 95%, 0.85–0.95; *p* = 0.0003), and intracranial hemorrhages (RR, 0.48; 95% CI, 0.39–0:59; *p* < 0.0001), despite the increase in gastrointestinal bleeding (RR, 1.25; 95% CI, 1.01–1.55; *p* = 0.04). In addition, reduced NOAC doses (dabigatran 110 mg BID or edoxaban 30 mg/day) showed similar results in terms of overall reduction of stroke and systemic embolism (RR, 1.03; 95% CI, 0.84–1.27; *p* = 0.74) and bleeding occurrence (RR, 0.65; 95% CI, 0:43–1:00; *p* = 0.05), despite the increase in ischemic stroke events (RR, 1.28; 95% CI, 1.02–1.60; *p* = 0.045).

The four registration trials differ according to the thromboembolic risk of enrolled populations. The highest rates of patients with CHADS_2_ score ≥3 were in the ROCKET-AF (87%) and ENGAGE (52%) trials, while only one-third of ARISTOTLE and RE-LY patients had CHADS_2_ ≥ 3 (30% and 32%, respectively) [36,37,38,39]. The incidence of major bleeding was similar in patients treated with rivaroxaban or warfarin in patients with CHADS_2_ score ≥3, whereas the incidence of major bleeding was higher in both dabigatran (110 mg or 150 mg), and the warfarin populations. This difference in the incidence of major bleeding appeared to be independent of the dose but rather was linked to the patient’s risk profile, as well as factors that may influence pharmacokinetics and pharmacodynamics (comorbidities, advanced age, HF, hepatic or renal insufficiency) [40]. 

During an extended follow-up (approximately four years), we found low rates of embolism (2.6%) and relatively few clinically relevant bleeding events (11.9%) (7 per 100 patient-years), thereby strengthening the conviction that NOACs, when adequately monitored, are safe and effective for elderly high-risk of bleeding patients, even in the presence of features like CKD. 

However, it is important to put our findings into context. Rohla et al. [41] conducted a large clinical study with 3156 patients receiving treatment with NOACs, mostly dabigatran. The mean age of these patients was 72 years, 40% being male, lower than in our study. The mean CHA_2_DS_2_-VASc score was 3.5, with 2.84% of the patients having major bleeding events. de Veer et al. [42] conducted a similar study, with 799 patients receiving NOACs, having a mean CHA_2_DS_2_-VASc score of 2.8. The rate of patients who had major bleeding events over a follow-up period of 1.7 years was found to be similar (bleeding events incidence rate of 6 per 100 patient-years) to our analysis. However, our study has a much longer duration of follow-up. 

Additionally, when comparing our findings with the above-mentioned pivotal NOAC randomized control trials (RCTs) [36,37,38,39], in which the mean CHADS_2_ score ranged from 2.1 to 3.5, the clinical complexity of our cohort is highlighted. The mean stroke risk of the patients followed at our anticoagulation unit was found to be higher than those reported in RCTs, which reinforces how real-world data is crucial to gain a more complete picture of NOAC advantages and disadvantages as they are used in everyday clinical practice. 

A retrospective real-world study conducted in three Spanish hospitals [43], which included 973 consecutive patients with nonvalvular AF who started treatment with DOACs, reported that during the follow-up period there were 101 clinically significant bleeding episodes (6.11/100 people/y), 47 major bleeding episodes (2.76/100 people/y), 40 significant gastrointestinal bleeding episodes (2.33/100 people/y), 25 major gastrointestinal bleeding episodes (1.46/100 people/y), five episodes of intracranial bleeding (0.29/100 people/y) and 102 deaths (5.85/100 people/y), 34 of which were of cardiovascular origin (1.95/100 people/y). Another study recently published [44] was an evaluation of a large real-world cohort of 30,401 patients ≥75 years, reported that the incidence (incidence/100 patient-years) of any hemorrhage was 8.3 for dabigatran, 12.6 for rivaroxaban, and 9.43 for apixaban, which is higher than our findings. 

A temporary interruption of NOACs was registered in 20.8% of our patients mainly due to medical interventions. This aspect should lead us to consider the important contribution of education of other specialists, namely the current recommendations on a drug suspension, and the situations in which hemorrhagic risk is reduced and suspension is not indicated.

While adequate NOAC dose prescription is required [45], such appropriateness remains to be further elucidated. 

The results from our cohort support—similar to findings from subgroup analyses of the pivotal RCTs with NOAC—that NOACs are an effective and safe option for elderly patients with AF. Careful consideration of pharmacological aspects and patient characteristics, is mandatory when applying guidelines to practice. Given the escalating complexity we face in the care of geriatric patients and other high-risk-for-bleeding populations, a structured follow-up, with judicious prescribing dosages and regular monitoring of renal function and drug compliance may reduce potential complications. 

### Study Limitations

The findings of this study have to be seen in the light of some limitations. Firstly, the observational nature of the study implies a risk of confoundings, such as the severity of pre-existing disease or the presence of comorbidities. As such, extraneous factors might have influenced the results. Unknown/unmeasurable confounders are inevitably present in observational studies, leading to residual confounding. Secondly, it is a retrospective study that includes a limited number of patients, conducted in the absence of a reference group treated with vitamin K antagonists.

Thirdly, given the real-world and, thereby, more limited set of the study, we cannot exclude the possibility that certain events were underestimated, namely minor bleeding. 

Finally, despite efforts to educate patients to improve compliance, we did not systematically measure adherence and, therefore, temporary drug interruption may not have been declared in all cases.

## 5. Conclusions

In this real-world observational study, the use of NOACs seems to be safe and efficacious among a high-risk of bleeding population. The rates of major bleeding and stroke were relatively low, and temporary NOACs discontinuation related to interventional procedures was common. Implementing a structured follow-up, with judicious prescribing, and an educational and drug compliance program may have the potential to prevent hemorrhagic and thromboembolic complications. 

## Figures and Tables

**Table 1:** Population clinical data.

Total Number of Patients		418
Age, y (mean ± SD)		77.88 ± 10.3
Age groups, *n* (%)	<65 years	38 (9)
	65–74 years	89 (21.2)
	≥75 years	289 (69.1)
Weight, Kg (mean ± SD)		73 ± 13.7
Male, *n* (%)		228 (54.5)
Comorbidities		
Hypertension		250 (55)
Diabetes mellitus		74 (17.7)
Heart failure		39 (9.3)
Prior stroke/TIA		65 (15.5)
Myocardial Infarction		8 (1.9)
COPD		60 (14.3)
Congenital heart disease		2 (4.1)
Previous pulmonary embolism		17 (4)
Use of NSAIDs, *n* (%)		16 (3.8)
Alcohol excess/abuse, *n* (%)		3 (0.7)
GFR (mL/min/1.73 m^2^), mean ± SD		59.7 ± 20.1
CHA_2_DS_2_-VASc score, mean ± SD		3.85 ± 1.4
CHA_2_DS_2_-VASc score, *n* (%)	≤1	18 (4.3)
	2	40 (9.5)
	≥3	359 (86)
HAS-BLED score, mean ± SD		1.85 ± 1.0

GFR: glomerular filtration rate.

**Table 2:** Type of NOAC and dosage (total *n* = 418 patients).

Type of NOAC	Dosage (mg)	*n* (%)
Apixaban (twice daily)	2.5	95 (22.6%)
	5	87 (20.8%)
Edoxaban (once daily)	15	1 (0.2%)
	30	57 (13.6%)
	60	79 (18.8%)
Dabigatran (twice daily)	75	3 (0.7%)
	110	9 (2.1%)
	150	4 (0.9%)
Rivaroxaban (once daily)	10	5 (1.1%)
	15	26 (6.2%)
	20	51(12.2%)

**Table 3:** Study endpoints.

Variable	Events no. (%)	Event Rate no./100 Patient-yrs.
Total Bleeding events (Major and Clinically Relevant Non-Major bleeding) *	50 (11.9)	7
Major Bleeding		
Total (any)	16 (3.8)	2.2
Transfusion	4 (0.95)	0.56
Decrease in hemoglobin > 2 g/dL	2 (0.47)	0.2
Critical Bleeding	10 (2.4)	1.4
Fatal Bleeding	0 (0)	0 (0)
Clinically Relevant Non-Major Bleeding	34 (8.1)	4.8
Stroke/TIA, *n* (%)	12 (2.6)	1.6
Venous Thromboembolic complications	2 (0.47)	0.28
Systemic arterial embolism	0 (0)	0 (0)
Hospital Admissions	59 (14.1)	8.3
All-cause Mortality, *n* (%)	36 (8.6)	5

* Bleeding complications are based on the definition of the International Society on Thrombosis and Hemostasis (ISTH) [23,24].

**Table 4:** Reasons for temporary discontinuation of NOAC therapy.

Reason for Discontinuation	n (%)
Biopsy	5 (1.1)
Surgery	50 (11.96)
Endoscopy	20 (4.7)
Trauma	5 (1.1)
Dental Extraction	7 (1.6)
Total	87 (20.8)

## Data Availability

The data presented in this study are available on request from the corresponding author. The data that support the findings of this study is not publicly available due to ethical restrictions as the information could compromise the privacy of research participants.
